# Regulation of Ferroptosis by Amino Acid Metabolism in Cancer

**DOI:** 10.7150/ijbs.64982

**Published:** 2022-02-07

**Authors:** Jian Yang, Xinyu Dai, Huanji Xu, Qiulin Tang, Feng Bi

**Affiliations:** Department of Medical Oncology, Cancer Center and Laboratory of Molecular Targeted Therapy in Oncology, West China Hospital, Sichuan University, Chengdu, Sichuan Province, 610041, China.

**Keywords:** amino acid metabolism, ferroptosis, cancer, combinatorial therapy

## Abstract

Ferroptosis, a new form of programmed necrosis characterized by iron-dependent lethal accumulation of lipid hydroperoxides, is associated with many human diseases. Targeting amino acid (AA) availability can selectively suppress tumor growth and has been a promising therapeutic strategy for cancer therapy. Compelling studies have indicated that AA metabolism is also involved in ferroptosis, closely regulating its initiation and execution. This manuscript systematically summarizes the latest advances of AA metabolism in regulating ferroptosis and discusses the potential combination of therapeutic strategies that simultaneously target AA metabolism and ferroptosis in cancer to eliminate tumors or limit their invasiveness.

## Introduction

Ferroptosis is defined as iron-dependent regulatory necrosis orchestrated by multiple molecular and metabolic pathways 1. Briefly, the regulatory mechanism of ferroptotic cell death mainly involves three parts: lipid peroxides (LPOs) generation, LPOs scavenging, and the repair of damaged plasma membranes 2. In the process of lipid peroxidation, the abundance of free iron, hydrogen peroxide, polyunsaturated fatty acids (PUFAs) and PUFA-rich phospholipids control the sensitivity to ferroptosis 2-3; The LPOs eliminating mechanisms mainly involves three pathways, including the cystine-glutamate antiporter transport system (system Xc^-^)/cysteine (Cys)/glutathione (GSH)/glutathione peroxidase 4 (GPX4), ferroptosis-suppressor-protein 1 (FSP1)/coenzyme Q (CoQ10), and GTP cyclohydrolase1 (GCH1)/tetrahydrobiopterin (BH4) axes 4. Some molecules such as tumor suppressor protein p53, nuclear factor erythroid 2-related factor 2 (NRF2), breast cancer gene 1 related protein 1 (BAP1), lncRNAs, etc. can act through regulating the activities of the defense system at the transcriptional or post-transcriptional level to control ferroptosis sensitivity 3-4; Ferroptotic cell death is booted by LPOs-induced membrane rupture. The endosomal sorting complex required for transport III (ESCRT-III) exerts a key part in repairing the damaged plasma membranes and control ferroptosis sensitivity 5. Since definition, ferroptosis has been implicated to be associated with the occurrence and development of various human diseases such as tumorigenesis, infection, immune diseases, neurodegeneration, and tissue ischemia-reperfusion injury 6-8. Especially for drug-resistant cancer cells, multiple experimental cancer models have demonstrated that they can be effectively killed by ferroptosis inducers, such as some small molecules and clinical drugs that target FSP1 and GPX4, deprive GSH or improve the iron pool. Nevertheless, smart cancer cells can always evolve alternative pathways to avoid this disadvantage. Therefore, exploring ferroptosis combination therapy might open up new therapeutic avenues for resensitizing cancer cells and eliminating drug-resistant clones. Studies have confirmed some synergy effects between ferroptosis and current cancer treatments, such as Roh's research 9 found that ferroptosis inducers can work synergistically with cisplatin to suppress the growth of head and neck tumors in mice; Wang's study 10 found that ferroptosis activators and anti-PD-L1 antibody nivolumab synergistically induce melanoma growth inhibition *in vitro* and *in vivo*; Moreover, studies have confirmed that ferroptosis inducers enhance radiotherapy sensitivity of melanoma 11, lung cancer and glioma 12-13, breast cancer 14 and nasopharyngeal carcinoma 15.

Studies about tumor metabolic reprogramming have been continually updated recently. As the amino acid (AA) metabolic network is the most complex and highly interconnected with other pathways, AA metabolic reprogramming has garnered considerable attention 16-17. AAs can not only serve as substrates for protein synthesis but also participate in energy production, macromolecular synthesis, signal transduction pathways, and the maintenance of cellular redox homeostasis 18-19. Emerging studies have revealed that AA availability is also involved in ferroptosis process and controls the susceptibility of tumors to ferroptosis. This review focuses on the regulation of ferroptosis by AA metabolism, and explores the potential of the combined strategies that target ferroptosis and AA metabolism to eradicate drug-resistant cancer.

## Amino acid metabolism in cancer

As the basis of protein synthesis and the intermediate metabolites that ignite other biosynthetic pathways, cancer cells with AA metabolic addiction have received an increasing attention in recent years 20. Studies have found that tumor cells usually rely on the supply of exogenous AAs, and this is not limited to essential amino acids (EAA) 21. Tumor cells have developed diverse mechanisms to maintain the abundant supply of AAs, including bidirectional transfer 22-23, micropinocytosis 24-25, or utilizing extracellular free AAs 26-27. Abnormal up-regulation of AA uptake and metabolism has been observed in many cancers 17. Therefore, it seems feasible to make tumor cells auxotrophic and then selectively lethal via interfering with AAs availability. Targeting AA metabolism such as interference with AA synthesis, degradation and transfer has been practiced in preclinical and clinical settings of cancer treatment, which provides numerous targets for anti-cancer drug development. For example, a study on colon cancer found 28 that targeting the methionine transporter solute carrier family 43A2 (SLC43A2) improved the expression of dimethylation at lysine 79 of histone H3 (H3K79me2) and signal transducer and activator of transcription 5 (STAT5) in T cells, thereby restoring T cell immunity. Another study 29 also found that elevating L-arginine concentrations promoted the survival ability of CD8+ T cells and their anti-tumor activity in mice. Werner's research 30 also confirmed that the arginine (Arg) transporter human cationic amino acid transporter-1 (hCAT-1) is a key component for activating efficient T cells to regulate the adaptive immune response in tumor immunity. These findings, including but not limited to, suggest that targeting cancer AA metabolism pathways may provide an immunotherapeutic approach. It is worth mentioning that the efficacy of metabolic inhibitor monotherapy may be limited, in that the metabolic changes of cancer cells exposed to AA deprivation may make the cells survive. Therefore, future prospects of AA deprivation therapy may involve combinations with other agents.

## Amino acid metabolism and ferroptosis

The trade of metabolites between ferroptosis and cancer cells is increasingly recognized as a critical aspect of tumor metabolism. Evidence has demonstrated that AA metabolism is critical for ferroptosis 31. Here we discuss the most prominent AAs involved in regulating ferroptosis and their possible regulatory mechanisms in cancer.

### Cyst(e)ine metabolism

Cys is a sulfur-containing proteinogenic AA whose free thiol group confers unique properties on protein functional sites 32. Cells have developed several mechanisms to keep a ceaseless supply of Cys. Tumor cells obtain Cys primarily via the uptake of cystine, a precursor of Cys, by system Xc^-^ 33. In addition, Cys could be directly assimilated via excitatory amino acid transporter 3 (EAAT3) and SLC1A4 transporter (ASCT1) 34-35. Endogenous Cys is synthesized by the trans-sulfuration pathway, which is mainly catalyzed by cystathionine γ-lyase (CSE) and cystathionine β-synthase (CBS) 36-39. Also, Cys could be obtained through autophagic breakdown of GSH and proteins 40. As a precursor of various biochemical processes, Cys actively participates in diverse metabolic pathways including GSH synthesis, protein s-cysteinylation, hydrogen sulfide (H_2_S) production, epigenetic regulation, and energy production 36.

Prior studies have indicated that Cystine/Cys depletion increases reactive oxygen species (ROS) production and induces ferroptosis, and certain cancers rely on Cystine/Cys metabolism to avoid ferroptosis 1, 32, 41. In the context of ferroptosis, Cys not only acts as a potent antioxidant by itself but also acts as an element of the major antioxidant GSH to maintain redox homeostasis 42-43. The reducing ability of the thiol group in Cys conferred its protective role against lethal ROS accumulation 44-45. Cys could be catabolized by two main pathways 36. In the first catabolic pathway, Cys could be degraded into compounds such as pyruvate and α-ketoglutarate (α-KG) by CBS and CSE. The former is metabolized to acetyl-coenzyme A (acetyl-CoA) and subsequently enters the tricarboxylic acid (TCA) cycle or is utilized to synthesize lipids, whereas the latter acts as an intermediate of TCA cycle or is used to synthesize glutamate (Glu) 46-49. Recently, it was reported that coenzyme A (CoA), a metabolite of Cys, cooperates with GSH to regulate ferroptosis in genetically engineered mice by controlling lipid metabolism 32, 50. However, the precise mechanism of anti-ferroptotic function of CoA is not discussed in the article. Studies have found that CoQ10, the derivative of CoA, also known as ubiquinone can be reduced by the oxidoreductase FSP1 to ubiquinol, a lipophilic radical-trapping antioxidant that halts the propagation of lipid peroxides 51-52. Therefore, CoA might act through generating CoQ10 to perform anti-ferroptotic function.

In the second catabolic pathway, cysteine dioxygenase (CDO1) catalyzes Cys transformation into taurine 53. CDO1 competes for Cys with glutamate-cysteine ligase (GCL), thereby shunting Cys used for GSH synthesis to taurine production 53-54. CDO1 has been demonstrated to decrease the intracellular level of GSH, thus enhancing ROS generation in cancer 55. Silencing or inactivating CDO1 helps to restore cellular GSH contents to avoid ROS and lipid peroxidation, which increases the resistance to ferroptosis, and promotes tumor proliferation 54-56. Therefore, restoring CDO1 function or increasing the expression of CDO1 protein in tumors may render tumor cells more vulnerable to ferroptosis, resulting in decreased tumor viability and growth. Although numerous studies indicate that CDO1 has tumor-suppressive properties, divergent evidence in glioblastoma 55 highlights the necessity for additional research to determine the true impact of Cys-derived compounds on cancer metabolic reconstruction.

Targeting the Xc^-^ system-mediated absorption of cystine is a classical approach to induce cell ferroptosis in basic studies. The clinical development of drugs targeting Cystine/Cys metabolic pathways to treat ferroptosis-related diseases may provide an avenue for future translation of this concept. It is still unknown whether human cancer is also susceptible to Cystine/Cys depletion-induced ferroptosis in clinic practice. A recent study demonstrates that the trans-sulfuration pathway is a direct advantage for cancer cells in maintaining redox equilibrium and evading ferroptosis. The trans-sulfuration pathway can produce endogenous Cys to synthesize GSH when cystine import is inhibited 57-59.

Cysteine persulfide (CysSSH) and cysteine polysulfides (CysSSnH, n > 1), which are Cys derivatives, have been proposed as powerful antioxidants and cytoprotective agents based on their superior nucleophilicity and reducibility 60. Study has found that CysSSnH can up-regulate the transcription of antioxidant genes, including the transcription of enzymes involved in GSH production to increase the level of GSH 61. In light of these findings, we reasonably speculate that CysSSH and CysSSnH are also very likely to participate in the ferroptotic regulation, despite the lack of direct reports on this currently. In this process, the key enzymes 3‐mercaptopyruvate sulfurtransferase (MPST), CBS, CSE and cysteinyl-tRNA synthetase (CARS) 62 that contribute to the production of CysSSH and CysSSnH may play an important role. Consistently, a screening of genome-wide siRNAs for ferroptosis inhibitors found that knockdown of CARS leads to the activation of trans-sulfuration pathway and ferroptosis resistance 58, 63.

**Figure 1:**
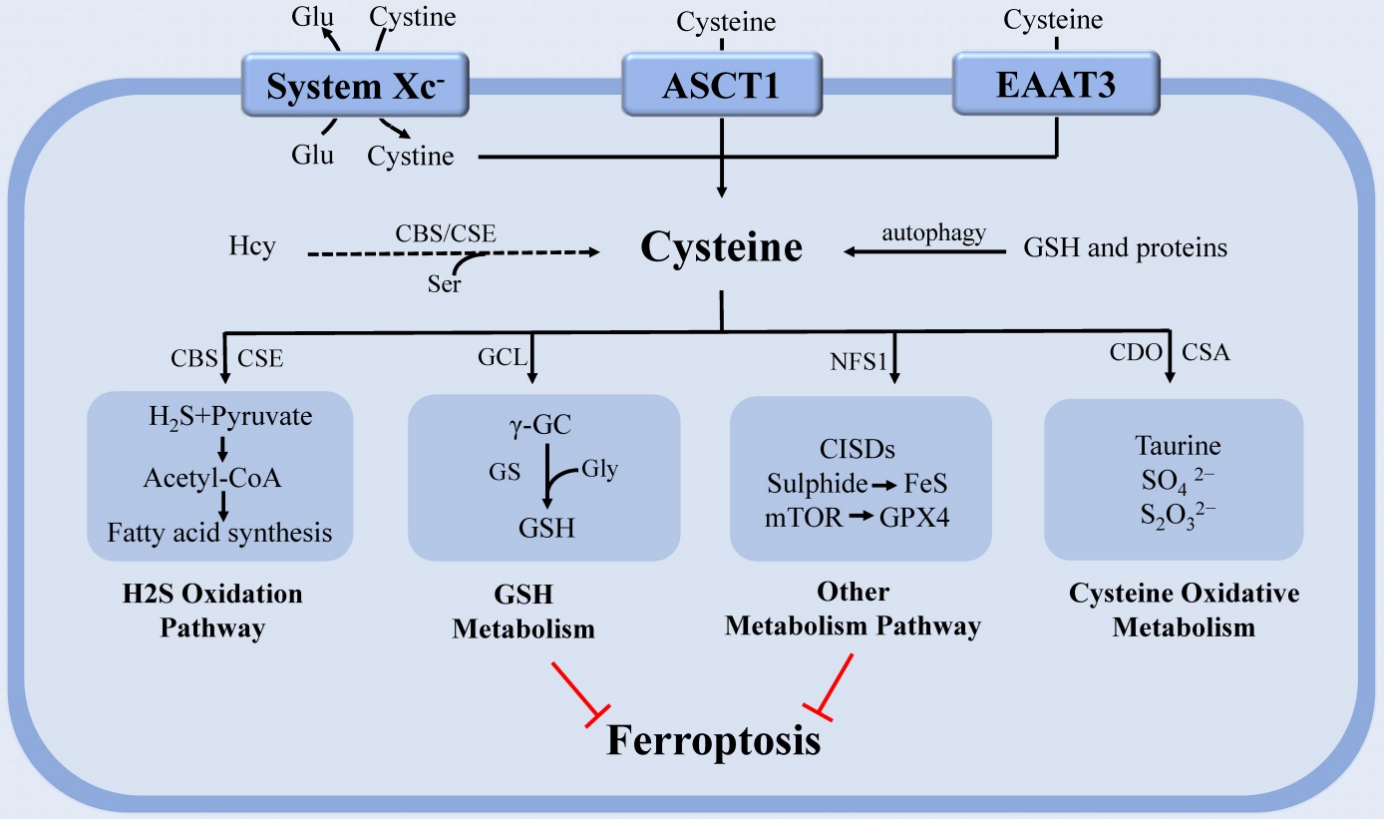
** The regulation of cysteine metabolism in ferroptosis.** Cysteine regulates ferroptosis through multiple pathways. In the H_2_S oxidation pathway, cysteine can be degraded into compounds which are used to synthesize fatty acids. PUFAs that undergo lipid peroxidation are involved in ferroptosis. The continuous supply of Fe-S clusters by mitochondrial NFS1 stopped the iron-starvation response. GCL can catalyze cysteine to form the antioxidant GSH. CDO, which catalyzes the conversion of cysteine to taurine, can compete with GCL for cysteine, thereby limiting GSH synthesis and promoting ROS. In addition, Cyst(e)ine could regulate ferroptosis independently of GSH by activating Rag-mTORC1-4EBPs signaling axis and promoting GPX4 protein synthesis. **Abbreviations:** system Xc^-^: cystine-glutamate antiporter transport system; ASCT1: solute carrier family 1A4 transporter: EAAT3: excitatory amino acid transporter 3; Glu: glutamate; Ser: serine; Gly: glycine; Hcy: homocysteine; PUFA: polyunsaturated fatty acid; GSH: glutathione; Fe-S: iron-sulfur; NFS1: cysteine desulfurase; GPX4: glutathione peroxidase 4; H_2_S: gasotransmitter hydrogen sulfide; ROS: reactive oxygen species; GCL: glutamate-cysteine ligase; CDO: cysteine dioxygenase; CBS: cystathionine β-synthase; CSE: cystathionine γ-lyase; GS: glutathione synthase; γ-GC: γ-glutamyl-cysteine; CSA: cysteine sulfinate.

Moreover, mitochondrial cysteine desulfurase (NFS1) can metabolize Cys and produce a sulfur carrier 36. NFS1 degrades Cys and releases sulfide to generate iron-sulfur (Fe-S) clusters, a cofactor of various essential proteins and enzymes in cells. Cells rely on increased NFS1 expression to maintain the continuous supply of Fe-S clusters when exposed to high oxygen concentration, as confirmed in metastatic or primary lung tumors 64. Although NFS1 has been shown to inhibit tumor growth via triggering the iron-starvation response, this state of iron deficiency can also protect cells from ferroptosis 36, 64. Therefore, targeting NFS1 seems to be a promising strategy to trigger ferroptosis for cancer treatment. Homma 65 also discovered a new mechanism by which Cys resists ferroptosis. This study discovered that Cys preservation conferred resistance of GSH depleted cells to ferroptosis through CDGSH iron sulphur domain-containing proteins (CISDs). CISDs exerted anti-ferroptotic function by suppressing free iron toxicity and the subsequent lipid peroxidation with the assistance of Cys. This study revealed a potential therapeutic strategy for eradicating ferroptosis-resistant cells by concurrently inhibiting GSH synthesis and CISDs activity (Figure 1).

Recent research discovered that cystine/Cys can activate Rag-mTORC1-4EBPs signaling axis and promote GPX4 protein synthesis to prevent cell ferroptosis in a GSH-independent manner. This study elucidated a regulatory mechanism that linked cystine/Cys availability to GPX4 protein synthesis and provided a new idea for using combinatorial therapy of ferroptosis inducers and mTORC1 inhibitors in cancer therapy 66. Cys metabolism is closely connected to that of glutamine (Gln) since serine (Ser), and glycine (Gly) can be derived from Gln and contribute to homocysteine (Hcy) and Cys syntheses through the one-carbon metabolism pathway 67-68. A study on Hcy found that it can promote ferroptosis via enhancing GPX4 methylation 69.

In conclusion, tumor resistance to ferroptosis caused by Cys deprivation can be reversed by concurrently targeting exogenous uptake and endogenous synthesis. It is necessary to explore additional and newer methods of targeting Cys metabolism to kill tumor cells more efficiently in the future. Cystine/Cys and Gln together form a metabolism network of AAs capable of providing the core metabolic pathways underlying key cancer processes. The production of Gln and its role in bioenergetics and signaling are addressed in the next section.

### Glutamine metabolism

Gln is the most abundant AA in the human body and the most critical non-toxic carrier in the ammonia cycle 70. In 2011, Hanahan and Weinberg 20 proposed the concept of tumor cell energy metabolic reprogramming in the new ten characteristics of tumor cells. Although glucose metabolic reprogramming plays a crucial biological significance in tumors, some tumor cells do not entirely rely on glucose metabolism to obtain energy 71. Subsequent studies have demonstrated that Gln is an important fuel, and also an important raw material for rapidly growing tumor cells 71-73.

After entering cell via the SLC1A5 (ASCT2), Gln mainly undergoes three metabolic routes 74: first, Gln is converted to Glu catalyzed by glutaminase (GLS), which is used to produce GSH together with Cys and Gly, and the generated GSH and NADPH can be used to maintain redox homeostasis 75; second, Gln acts as a raw material to provide precursors for synthesizing various nucleotides, AAs, proteins, lipids and other biologically important molecules 76-77; third, Gln enters into the mitochondria and is converted into α-KG catalyzed by GLS and glutamate dehydrogenase (GDH). α-KG participates in the TCA cycle to produce adenosine triphosphate (ATP) and replenish TCA cycle intermediates 77-79. While being a non-essential amino acid (NEAA), Gln is essential for rapidly proliferating cells, such as cancer cells 80. *In vivo* experiments in hepatomas and hepatic fibrosarcoma revealed that cancer cells consume Gln ten times faster than normal hepatocytes 81. In this context of undersupply, many cancer cells reprogram their metabolism pathways to take up more Gln by upregulating Gln transporters or enhancing the expression or activity of Gln key metabolic enzymes 82, shifting Gln utilization from catabolic to anabolic, and facilitating the biosynthesis of macromolecules and organelles required for assembling new cells 20, 83-84.

Gln metabolism is tightly linked to ferroptosis regulation 85. To date, the precise physiological function of Gln in ferroptosis remains unknown. Gao's study 86 found that Gln may be an inducer of ferroptosis, fueling ferroptosis through its specific metabolism, glutaminolysis. Glutaminolysis refers to a series of intracellular biochemical reaction processes driven by Gln, in which Gln is converted to Glu, aspartic acid, carbon dioxide, pyruvate, lactic acid, alanine, citric acid, and other products catalyzed by key enzymes, such as GLS, GDH, and aspartate aminotransferase 2 (GOT2) and subsequently used as a fuel for TCA cycle and lipid biosynthesis 86-87. Gao's research team further demonstrated that L-Gln, but not D-Gln, is responsible for this type of cell death 86. When lack of Gln or glutaminolysis is inhibited, cystine starvation or preventing cystine import cannot induce ferroptosis, ROS accumulation, or lipid peroxidation 86, 88. Meanwhile, high-dose extracellular Gln alone cannot induce ferroptosis. Ferroptosis can be induced only when Gln is available, accompanied by cystine deprivation 86.

Consistent with previous research, deprivation of cystine or Cys or inhibition of the system Xc^-^ induced ferroptosis by depleting cellular GSH and accordingly increasing ROS, and concurrently accelerated the death process through Glu accumulation, the glutaminolysis product 3, 89. The glutamate-cysteine ligase catalytic subunit (GCLC) inhibits ferroptosis by participating in the first step of GSH synthesis. However, Yun et al. 89 found that GCLC plays a GSH-independent, non-classical role in preventing ferroptosis by regulating Glu pool under cystine deprivation. GCLC mediates γ-glutamine peptide synthesis, limits Glu accumulation, and thus protects against ferroptosis. In addition, GCLC activity is regulated by NRF2, a pivotal transcriptional regulator. This study indicates how cells save themselves under cystine or Cys starvation conditions, but it does not explain how Glu accumulation promotes ferroptosis.

**Figure 2:**
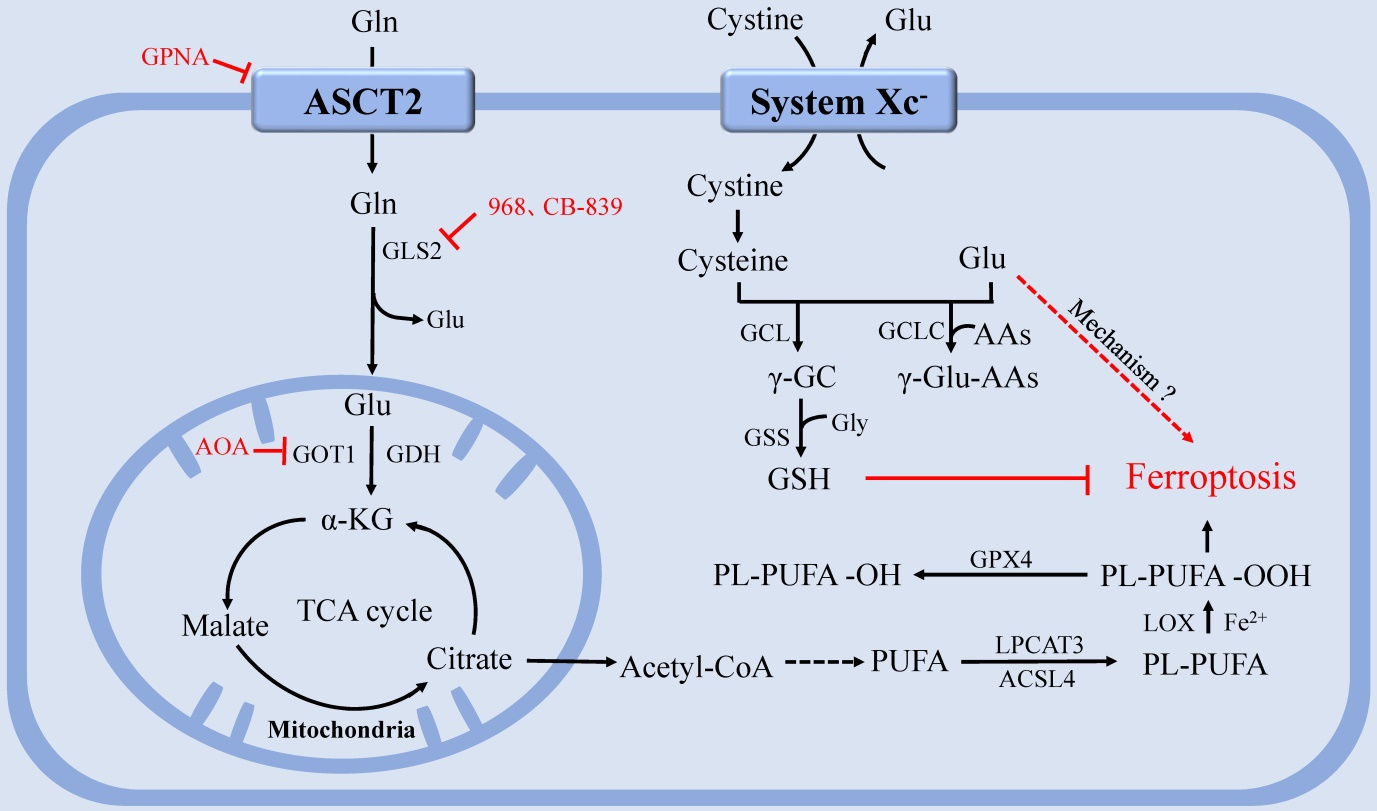
** The regulation of glutamine metabolism in ferroptosis.** After entering the cell via the ASCT2, Gln can be degraded and provide a precursor for TCA and PUFAs biosynthesis. System Xc^-^ inputs cysteine to synthesize GSH and exchange Glu at the same time. GPX4 utilizes GSH to eliminate lipid peroxides that participate in ferroptosis. GCLC maintains the Glu pool homeostasis under cystine starvation by mediating the synthesis of γ-glutamine peptide, thereby limiting the accumulation of Glu and protecting against ferroptosis. **Abbreviations:** ASCT2: solute carrier family 1A5 transporter; Gln: Glutamine; Glu: glutamate; GLS: glutaminase; GDH: glutamate dehydrogenase; GOT1: aspartate aminotransferase 1; TCA: tricarboxylic acid; PUFAs: polyunsaturated fatty acids; system Xc^-^: Cystine-glutamate antiporter transport system; GSH: glutathione; GPX4: glutathione peroxidase 4; GCLC: glutamate-cysteine ligase catalytic subunit.

The function of glutaminolysis in ferroptosis could also be explained by the discovery that α-KG, a product of glutaminolysis, could substitute Gln to function in Cys deprivation-induced lipid ROS accumulation and ferroptosis 75, 88. Furthermore, TCA metabolites downstream of α-KG, including malate, fumarate, and succinate, were all able to substitute the function of Gln in regulating lipid ROS accumulation 87. Notably, compared with Gln status, glucose status has a greater impact on TCA cycle metabolites upstream of α-KG, such as citrate, as the role of glutaminolysis in TCA cycle is primarily to replenish TCA cycle intermediates 79, 88. Interestingly, it has been demonstrated that ferroptosis requires mitochondrial GLS2 rather than cytosolic GLS1, although both enzymes catalyze glutaminolysis 86 (Figure 2).

At present, studies on Gln starvation therapy have confirmed that Gln transporter inhibitor GPNA 89, GLS inhibitor compound 968 90, CB-839 91-92, and transaminase GOT1 inhibitor aminooxyacetic acid (AOA) 76 can significantly inhibit ferroptosis. Gene interference or pharmacological inhibition of ASCT2 has been shown to decrease the growth of prostate cancer 93, gastric cancer 94, and triple-negative breast cancer 95. However, since compensatory responses are triggered, blocking a single Gln transporter is insufficient to prevent tumor growth 17. Therefore, blocking the uptake or degradation of Gln in multiple ways may become a more viable therapeutic strategy for ferroptosis-associated diseases.

### Branched-chain amino acids metabolism

Branched-chain amino acids (BCAAs), including leucine, valine, and isoleucine, are a subclass of EAAs whose metabolism has been linked to specific cancer phenotypes 96. BCAA metabolism changes can both affect intrinsic cancer properties of cells and represent systemic metabolic changes correlated with certain cancers 96. After dietary intake, BCAAs are transported by L-type amino acid transporters (LATs) into the cell and are catabolized by highly reversible branched-chain amino acid transaminases (BCATs) 97-98. BCAT exists in two isoforms, BCAT1 and BCAT2 98. BCAT2 is located in mitochondria and is ubiquitously expressed, while BCAT1 is located in cytosol, and its expression is restricted to certain organs, such as the brain 99.

Both BCAT1 and BCAT2 are highly active and reversible enzymes that catalyze all three types of BCAAs and their corresponding branched-chain keto acids (BCKAs) 100. BCAT produces Gln and designated BCKA by transferring nitrogen to a-KG, and the produced Glu can be used to support AA and nucleotide pools 101. In addition, the generated keto acids are further metabolized by several steps to acetyl-CoA and/or succinyl-CoA, which were used to replenish TCA cycle intermediates and participate in synthesizing fatty acids 98. BCAAs at high concentrations have been demonstrated to increase ROS production and mitochondrial dysfunction by activating Akt-mTOR signaling pathway 102. However, a recent study demonstrated that BCAT1 could mediate EGFR tyrosine kinase inhibitors (TKI) resistance by producing GSH to counteract ROS accumulation 103. Furthermore, another study also identified BCAT2 as a novel suppressor of ferroptosis, whose activation of ectopic expression could specifically antagonize system Xc^-^ inhibition and protect liver cancer and pancreatic cancer cells from ferroptosis. Therefore, BCAT2 could serve to predict the responsiveness of cancer cells to the ferroptosis-inducing therapy 104. Together, reprogramming of BCAA metabolism could change mitochondrial functions as well as gene expression, redraw other metabolic pathways, and change the levels of essential metabolites, including Glu, α-KG, BCAAs and ROS, therefore elevating cancer cell proliferation and enhancing drug resistance capacity 98.

### Tryptophan metabolism

As EAA, tryptophan (Trp) and its metabolites perform various nutritional and physiological roles and are intimately linked to regulating cancer, neurodegeneration, and other diseases 105. As a building block of proteins, Trp is required for cell growth and maintenance. As a neurotransmitter and signaling molecule, Trp is required to transmit organismal responses to dietary and environmental signals 106. The content of internal free Trp depends on the external food intake and the activity of Trp metabolic pathway. Among them, more than 95% of free Trp functions as a substrate to participate in the kynurenine (Kyn) pathway, which produces various metabolites with different biological activities. The rate-limiting step in the Kyn pathway is performed by three enzymes indoleamine-2,3-dioxygenase 1 (IDO1), IDO2, and tryptophan-2,3-dioxygenase (TDO). They consume Trp by converting Trp into N-formylkynurenine (NFK), which accordingly has a fundamental impact on cellular function and survival 106. In cancer, aberrant activation of IDO1 and TDO results in suppression of anti-tumor immunity 107. Therefore, drugs combining IDO1 inhibitors and immune checkpoint inhibitors have been developed for enhancing anti-tumor immunity 108. Although there are few reports about the metabolites or key enzymes of the Kyn pathway involved in regulating ferroptosis, a recent study revealed that IDO1 deficiency contribute to ferroptosis resistance by activating the expression of SLC7A11, (xCT), while IDO1 overexpression could exacerbate the classical ferroptosis events 109.

**Figure 3:**
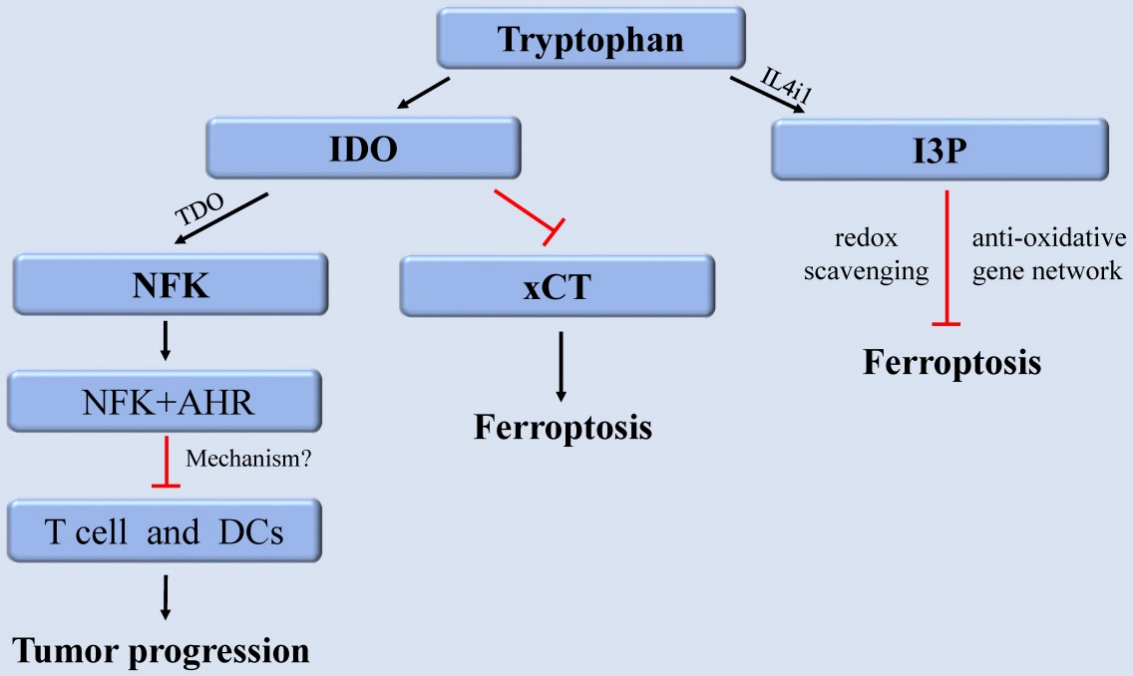
** The regulation of tryptophan metabolism in ferroptosis.** Trp can be converted into NFK under the catalysis of IDO and TDO to regulate tumor growth. IDO aggravates ferroptosis by inhibiting xCT. In addition, Trp can also be metabolized to I3P under the catalysis of IL4i1 to prevent ferroptosis. **Abbreviations:** Trp: tryptophan; IDO: indoleamine-2,3-dioxygenase; TDO: tryptophan-2,3-dioxygenase; NFK: N-formylkynurenine; AHR: aryl hydrocarbon receptor; xCT: solute carrier family 7 member 11; I3P: indole-3-pyruvate; IL4i1: nterleukin 4 induction 1.

Furthermore, recent research discovered that Trp metabolite, indole-3-pyruvate (I3P) can regulate ferroptosis 110. Trp can be metabolized to I3P catalyzed by interleukin 4 induction 1 (IL4i1), a FAD-dependent oxidoreductase that metabolizes AAs and is linked to immune suppression in cancer. I3P protects against ferroptosis in at least two distinct ways: by directly scavenging free radicals and activating anti-oxidative stress pathways 110. I3P can up-regulate the protein levels of SLC7A11, NQO1, ATF4, CYP1B1, and the AKR1C family at the transcriptional level. These proteins are all involved in oxidative stress response, which can induce cell resistant to ferroptosis-induced oxidative damage. Furthermore, compared with the metabolites derived from the catalyzation of other AAs by IL4il, I3P exhibited the highest radical scavenging potency against the exogenous stable radical diphenyl-2-pyridohydrazine (DPPH). Therefore, I3P might also be able to trap lipid peroxyl radicals directly to prevent ferroptosis 110. Given that IL4i1 activates anti-ferroptosis pathways through I3P and possibly other indoles, we speculate that blocking IL4i1-mediated Trp metabolism could be a useful anti-cancer therapeutic strategies (Figure 3).

### Other amino acids metabolism

Other AAs, such as Arg, Ser, Gly, lysine (Lys), etc., also play a critical role in cell metabolism and cancer development. Arg could serve as a precursor of many biomolecules, such as creatine, nitric oxide, polyamines, proteins, and other AAs 10. In recent years, Arg starvation has become a potential and novel clinical strategy for cancer therapy 111-112. There is currently a lack of research on Arg metabolism in regulating ferroptosis. But numerous evidences indicate that Arg consumption is intimately linked to ROS production 113. A study exploring the effect of Arg on lipopolysaccharide (LPS)-induced oxidative stress found that the intracellular GPX content was significantly increased after the addition of Arg, accompanied by a decrease in ROS and malondialdehyde (MDA) content, and this process was realized at least partially through arginase-1 signaling pathway 114. These findings indirectly imply that Arg metabolism is likely to participate in the regulation of ferroptosis.

The *de novo* synthesis of Cys fails to perform without considering Ser and Gly in the one-carbon pathway 16. Activating Ser synthesis pathway is directly correlated with GSH synthesis 115, as Ser itself participates in Cys synthesis, and serine-derived Gly is a component of GSH 19,116. Along with its anabolic function in nucleotide synthesis and protein translation, Ser catabolism in mitochondria is crucial to maintain NADPH production and redox balance 117. Therefore, it can be inferred that Ser and Gly might be able to regulate ferroptosis by participating in GSH synthesis and redox reactions. Many studies have noted that tumors, such as melanoma, breast cancer, lung cancer, and Ewing's sarcoma could activate the serine-glycine biosynthetic pathway to support their growth 118-121. The *de novo* synthesis of serine-glycine has been established as an important factor in tumorigenesis. And the inhibition of serine-glycine biosynthesis could interfere with their redox state, leading to the ROS accumulation and cell death 121. Nevertheless, there is still a lack of direct evidence that Ser or Gly can regulate ferroptosis.

A recent study showed that Lys was also involved in the regulation of ferroptosis, which found that L-lysine α-oxidase, a member of the L-amino acid oxidase family, activates ferroptotic signal by catalyzing the oxidative deamination of L-lysine and ROS production 122. The most updated situation of key enzymes/transporters or genes of AA metabolism that possibly participates in the regulation of ferroptosis are presented in Table 1.

## Perspectives and Conclusions

Since the concept of “ferroptosis” was defined, people have continuously explored its therapeutic potential in lipid peroxidation diseases. In fact, it has been confirmed in many cancers that they are more susceptible to this iron-catalyzed necrosis. However, working hard to develop tolerance and escape various forms of death seems to be the eternal hallmark of cancer cells, and ferroptosis is no exception. The sensitivity of cell to ferroptosis is correlated with numerous biological processes, including AA metabolism.

As basic nutrients and energy sources, AAs contribute significantly to tumor proliferation and homeostasis by acting as intermediates in the metabolism of glucose, lipids and nucleotides. As new functions continue to be discovered, AA metabolism has exerted an increasing part in cancers. Numerous cancers exhibit a high requirement for specific AAs acquired exogenously or released endogenously. Thus, specific AA deprivation could shut down nutrient supply, resulting in AA starvation and cell death. Some AA starvation therapies designed according to the high demand of tumor cells for specific AAs have been introduced into clinical practice or are undergoing clinical evaluation, such as Arg, asparagine, Lys, methionine, phenylalanine, tyrosine and so on 130. However, although AA depletion therapy has broad applicability in cancer treatment, metabolic inhibitor monotherapy may render cancer cells drug-resistant due to compensatory activation and cross-interference of metabolism pathway or reactivation of the silenced genes.

Targeting AA metabolism is currently moving towards combinatorial studies based on the synergistic drug interactions, such as the study of combining immunotherapy and metabolic inhibitors. Although the current research on the regulation of ferroptosis by AA metabolism is limited, the combinatorial targeting of AA metabolism and ferroptosis has shown great promise in cancer treatment. While there are still some thorny issues for this mode of therapy that urgently need to be further studied. For example, it is necessary to clarify the metabolic dependence in specific cancer type, so as to select an appropriate AA target. Attention should also be paid to the crosstalk of AA metabolism between cancer cells and immune cells as well as normal cells in the surrounding environment, as ferroptosis may affect cancer immune surveillance in a dual way. A full understanding of metabolic flexibility and diversity of ferroptosis in cancers will help better guide drugs usage and cancer treatment.

## Figures and Tables

**Table 1:** Key enzymes/transporters or genes that may participate in the regulation of ferroptosis in amino acid metabolism

Compound/Drug	Target	Mechanism	Phase (status)	Reference
sorafenib	system Xc^-^	Prevents cystine import, causes GSH depletion	Approval	123
erastin	system Xc^-^	Prevents cystine import, causes GSH depletion	Phase I	1
sulfasalazine	system Xc^-^	Prevents cystine import, causes GSH depletion	Approval	1
BSO	GCL	Inhibits GCL, inhibits GSH synthesis.	Phase I	124
artesunate	glutathione S-transferase	Inhibits glutathione S-transferase, causes GSH depletion	Approval	125
cyst(e)inase	Cyst(e)ine	Induces cyst(e)ine depletion		32
CB-839, BPTES 968	GLS	Inhibits the conversion of Gln to Glu	Phase I/II	87, 91
AOA	GOT1	Inhibits the conversion of Glu to a-KG		87
ADI-PEG20	Arginine	degrades and consumes Arg	Phase III	126
CB-1158	Arginase	Inhibiting arginase	Phase II	127
GPNA, Tamoxifen Raloxifene	ASCT2	Inhibits glutamine uptake		91, 128
Gene	Protein	Mechanism	Phase (status)	Reference
CDO1	cysteine dioxygenase	enzyme that catalyzes the conversion of cysteine to taurine and reduces GSH synthesis		53
CISD1	CDGSH iron-sulfur domain 1	Inhibits mitochondrial iron transport into the matrix		129
SLC7A11	Solute carrier family 7 member A11, xCT	a component of system Xc^-^, requires for cystine import		82
CARS	cysteinyl-tRNA synthetase	knockdown causes increased transsulfuration pathway activity, and resistance to ferroptosis		58
NFS1	cysteine desulfurase	enzyme involves in synthesizing iron-sulfur clusters using sulfur from cysteine		64
SLC1A5	solute carrier family 1 member 5	amino acid transporter feeding glutaminolysis		86
GCLC	glutamate-cysteine ligase catalytic subunit	enzyme involves in GSH synthesis		124
BCAT2	branched-chain amino acid transaminase-2	activation could antagonize system Xc^-^ inhibition and protect from ferroptosis.		104
I3P	indole-3-pyruvate	scavenges free radicals and activates anti-oxidative stress pathways		110
LO	L-lysine α-oxidase	enzyme catalyzes the oxidative deamination of L-lysine to activate ferroptosis		122
